# Draft genome sequence of *Paenibacillus* sp. strain Dod16 isolated from *Glossina palpalis palpalis* in Cameroon

**DOI:** 10.1128/mra.01327-25

**Published:** 2026-03-06

**Authors:** Youssouf M. Mfopit, Judith S. Engel, Rolf Nimzyk, Andrea Schaffrath, Petra Berger, Daniel M. Achukwi, Mohammed Mamman, Emmanuel O. Balogun, Mohammed N. Shuaibu, Junaidu Kabir, Barbara Reinhold-Hurek, Sørge Kelm

**Affiliations:** 1 Institute of Agricultural Research for Development 231172, Yaounde, Cameroon; 2 Department of Biochemistry, Ahmadu Bello University 58989, , Zaria, Nigeria; 3 Africa Centre of Excellence for Neglected Tropical Diseases and Forensic Biotechnology (ACENTDFB), Zaria, Nigeria; 4 MARUM-PANGAEA, University of Bremen 9168, Bremen, Germany; 5 Centre for Biomolecular Interactions Bremen, University of Bremen 9168, Bremen, Germany; 6 TOZARD Research Laboratory, Bamenda, Cameroon; 7 Department of Veterinary Pharmacology and Toxicology, Ahmadu Bello University 58989, Zaria, Nigeria; 8 Department of Veterinary Public Health and Preventive Medicine, Ahmadu Bello University 58989, Zaria, Nigeria; Portland State University, Portland, Oregon, USA

**Keywords:** *Paenibacillus *sp., genome, tsetse microbiota, *Glossina*

## Abstract

Little is known about *Paenibacillus* species’ role in tsetse microbiota influencing vector competence. We report the draft genome of *Paenibacillus* sp. strain Dod16 isolated from the Tsetse fly midgut in Dodeo, Cameroon. The genome is 8,067,571 bp in length, with 8,150 predicted coding sequences and a G+C content of 48.2%.

## ANNOUNCEMENT

African trypanosomiasis, transmitted by tsetse flies (*Glossina* spp.), continues to threaten humans and livestock across sub-Saharan Africa (1). Tsetse flies harbor complex gut microbial communities that influence their vector competence (2). *Paenibacillus* sp. are rod-shaped, gram-positive, environmentally acquired bacteria (3). Some species produce antimicrobial compounds or enzymes with potential biological activity that could modulate insect-microbiota interactions. Here, we report the genome sequence of a *Paenibacillus* strain, Dod16.

Tsetse flies collected in December 2017 with biconical traps in Dodeo, Cameroon (N7.440560, E12.00803), were dissected aseptically. Gut contents were cultured in Maramorosch and Mitsuhashi insect medium (4), supplemented with 20% fetal calf serum. Single bacterial colonies were obtained by repeated streaking and incubation at 30°C under anaerobic conditions (Oxoid AnaeroGen, Thermo Scientific). DNA was extracted using bead-beating (5), and the 16S rRNA gene was amplified (6) and sequenced for genus identification. Genome sequencing was performed on an isolate selected based on positive sialidase activity and clear taxonomic assignment by 16S rRNA analysis. Genomic DNA was purified using the phenol-chloroform method (7), with concentration and purity assessed by NanoDrop spectrophotometry and integrity confirmed by electrophoresis.

Extracted DNA was sheared by ultrasonication on Covaris M22. DNA library preparation was performed using an Illumina TruSeq DNA PCR-free kit. A quality check was performed on an Agilent Bioanalyzer 2100 using a high-sensitivity DNA kit, and DNA concentration was analyzed by qPCR using the NEBNext Library Quant Kit (New England Biolabs). Sequencing was done on Illumina MiSeq using the MiSeq Reagent Kit v2 Nano with 150 bp paired-end. There was a total of 336,263 reads, with a quality score of 89.9% >= Q30.

Reads were trimmed using Trimmomatic (Version: v.0.39), and sequences were assembled using SPAdes v3.15 (8). Annotation was performed using the Rapid Annotation System Technology (RAST) server (9). TYGS v403 was used for whole genome-based taxonomic analysis and digital DNA-DNA hybridization (dDDH; formula d4) (10). All tools were run with default parameters unless otherwise specified.

BLAST analysis against the NCBI database showed the 16S rRNA gene sequence shared 99.7% identity with *Paenibacillus lautus* strain MER_TA_68 (KT719472.1). The sequencing coverage was 31.02×. The genome assembly was found to be 98.44% complete based on CheckM (v1.2.3) analysis. The draft genome sequence of strain Dod16 consisted of 156 contigs (N50 = 99,616; L50 = 25) and at least 8,067,571 bases in length, with an average G+C content of 48.2%. The genome contained 8,150 coding sequences and 78 structural RNA genes. The coding sequences were classified into 318 SEED subsystems, of which the most abundant were aa and derivatives (*n* = 302), carbohydrates (*n* = 298), and protein metabolism (*n* = 228). Of the 8,150 coding sequences identified, 18% were featured in SEED subsystems, while 82% were not.

*Paenibacillus* sp. Dod16 does not belong to any species found in the TYGS database, suggesting a potential new species. Dod16 had the highest dDDH_4_ of only 47.6% with *Paenibacillus lautus* NBRC 15,380 (lower than the cutoff point of 70%).

These data provide a genomic resource toward exploring alternative microbiome-based strategies for trypanosomiasis control.

## Data Availability

The Whole Genome Shotgun project of *Paenibacillus* sp. Dod16 has been deposited in DDBJ/ENA/GenBank under accession number JBNDYG000000000. The version described in this paper is the first version. The BioProject accession number is PRJNA1250976 and the BioSample accession number is SAMN42321958. The raw reads were deposited in SRA database under accession number PRJNA1250976.
